# Supplementary material for: Lung Transplant Immunomodulation with Genetically Engineered Mesenchymal Stromal Cells—Therapeutic Window for Interleukin-10
Source: Cells. 2024 May 17;13(10):859. doi: 10.3390/cells13100859 (PMC11119666; doi:10.3390/cells13100859)
Supplement: Supplementary file 1 [file cells-13-00859-s001.zip › cells-2998618-supplementary.pdf]

Supplementary Material

Lung transplant immunomodulation with genetically engineered mesenchymal stromal cells – therapeutic window for interleukin-10

Antti I. Nykänen, MD, PhD,[1,2] Andrea Mariscal, MD, PhD,[1,2,3,4] Allen Duong, BSc,[1,2] Aadil Ali, PhD,[1,2] Akihiro Takahagi, MD,[1] Xiaohui Bai, MSc,[1] Guan Zehong, MSc,[1] Betty Joe, BSc,[1] Mamoru Takahashi, MD, PhD,[1] Manyin Chen, MD,[1,4] Hemant Gokhale, MD,[1,4] Hongchao Shan, MD,[1,4] David M. Hwang, MD, PhD,[5] Catalina Estrada, PhD,[6] Jonathan Yeung, MD, PhD,[1,2,3,4] Tom Waddell, MD, PhD,[1,2,3,4] Tereza Martinu, MD, MHS,[1,2,3,7] Stephen Juvet, MD, PhD,[1,2,3,7] Marcelo Cypel, MD, MSc,[1,2,3,4] Mingyao Liu, MD, MSc,[1,2] John E. Davies, BDS, DSc, PhD,[6,8] Shaf Keshavjee, MD, MSc[1,2,3,4]

1. SUPPLEMENTARY MATERIALS AND METHODS

1.1 Experimental Design

Human umbilical cord perivascular mesenchymal stromal cells (MSCs) were genetically engineered for augmented human IL-10 production (MSC^{IL-10}) by adenoviral transduction and cryopreserved 48 hours after transduction. IL-10 secretion of the cryopreserved $MSCs^{IL-10}$ was confirmed *in vitro*. Pig double lungs subjected to 24-hour cold ischemia were connected to clinical-grade EVLP for 6 hours and randomized to control (n=7), or to receive $20x10^6$ (n=5) or $40x10^6$ cryopreserved MSC^{IL-10} (n=6) through the pulmonary artery in a blinded manner. Lung function parameters, perfusate, lung tissue and bronchoalveolar lavage (BAL) samples were collected during EVLP. After EVLP, the left lung was transplanted into a recipient pig that received methylprednisolone and cyclosporine A background immunosuppression. Three days after transplantation, graft evaluation was performed. Blood, tissue and BAL samples were collected for biochemical, histological and immunological analysis. Detailed n-values for each phase of the study are given in Table S1 and in the respective Figure Legends.

1.2 Generation of $MSCs^{IL-10}$

Human umbilical cord perivascular MSCs were isolated and expanded, genetically modified, and bench-tested as described previously in detail.[1] Briefly, human umbilical cords from full-term, consenting donors undergoing caesarean section at Mount Sinai Hospital (Toronto, Canada) were sourced and extracted, with approval from ethics boards

at both the University of Toronto and Mount Sinai Hospital Research Centre for Women's and Infants' Health (RCWIH), using a proprietary methodology and provided by Tissue Regeneration Therapeutics (TRT) Inc. (Toronto, Canada). The perivascular Wharton's jelly tissue was stripped from the vessel walls, minced, and seeded in fibronectin-coated culture flasks, in Lonza TheraPEAK™ MSCGM-CD serum-free medium (Cedarlane, Burlington, Canada) for 14 days to isolate cells; adherent cells were dissociated with TrypLE Express (ThermoFisher Scientific, Waltham, MA). After centrifugation, the cells were washed, culture expanded to P1, cryopreserved as a master cell bank, and later thawed in RoosterNourish™ MSC-XF medium (RoosterBio Inc., Frederick, MD) for expansion to P2 and stockpiled in liquid nitrogen.

MSCs were genetically engineered by exposing the cells to replication-incompetent recombinant serotype 5 adenovirus vectors with CMV promoter-driven expression of human IL-10 (including a FLAG tag; Vigene Biosciences, Rockville, MD; # VH869610). After 24 hours of incubation at 37°C, 5% CO_2, cells were washed, fresh growth medium was added, and the engineered $MSC^{IL\text{-}10}$ were harvested 48 hours later and cryopreserved in liquid nitrogen for future use.

1.3 In vitro evaluation of MSCs

Cryopreserved $MSCs^{IL\text{-}10}$ were evaluated after thawing for IL-10 secretory activity *in vitro* by seeding $54x10^3$ $MSCs^{IL\text{-}10}$ in 12 mm Transwells with 3.0 μm pore membrane inserts (Corning Life Sciences, Corning, NY) in a total of 2 mL of EVLP perfusate solution per well at 37°C, 5% CO_2. Samples were taken from the bottom compartment at 5 and 30

min and at 1- 5 hours. ELISA was performed to determine human IL-10 concentration (Human IL-10 DuoSet ELISA, R&D Systems Inc, Minneapolis, MN).

1.4 EVLP

Pig lungs were harvested as described previously,[2] donor blood and spleen samples were collected, and the inflated double lung was stored at +4°C in a cold room for 24 hours. After cold storage, the lungs were connected to a clinical-grade EVLP as described previously[3] for 6 hours. Briefly, the atrial cannula was sutured to the donor atrium, pulmonary artery cannula was connected to the main pulmonary artery, a 7.5 Fr endotracheal tube was inserted in the trachea, and the lung was flushed retrogradely with low potassium dextran solution (Perfadex, XVIVO Perfusion, Gothenburg, Sweden). The EVLP circuit was primed with 1,500 ml of EVLP perfusate (LPD2A, United Therapeutics, Silver Spring, MD) supplemented with heparin 10,000 IU, methylprednisolone 1 g and cefazolin 1 g. During the initial hour, EVLP flow was increased incrementally to 40% of the calculated total cardiac output, temperature was gradually increased, and ventilation started at 32°C with tidal volume 7 ml/kg, PEEP 5 cmH_2O, FiO_2 0.21 and rate 7 per min. Hourly lung recruitment was performed by two 3-second inspiratory holds with a peak airway pressure of 25 mmHg. Lung function parameters and perfusate samples were taken every hour after increasing FiO_2 to 1.0. Perfusate pH, pCO_2, pO_2, glucose and lactate were immediately analyzed with a point-of-care system (RAPIDPoint 500 Blood Gas System, Siemens Healthineers, Erlange, Germany) and complete, and supernatant and cell fractions, of perfusate were stored at -80°C and -150°C (cell fraction). The EVLP

protocol consisted of adding 100 ml of fresh perfusate to the circuit at 2 and 4 hours. Right lower lobe bronchoalveolar lavage (BAL) with 30 ml of saline was performed at 1 hour, before MSC^{IL-10} administration, and at 6 hours using a flexible fiber-optic bronchoscope, and complete, and supernatant and cell fractions were cryopreserved. Right lower lobe lung biopsies were taken before EVLP start, and at 3 and 6 hours. After EVLP, the lung was cooled down to +10°C, flushed antegrade with Perfadex® solution, the left lung was used for transplantation, and lung samples were taken from the right upper and lower lobe superficial (including parietal pleura) and deep areas (excluding parietal pleura). Lung tissue sample processing consisted of 1) snap freezing in liquid nitrogen, tissue homogenization, protein isolation[4] and protein concentration measurement (Pierce BCA Protein Assay Kit, Thermo Fisher Scientific), 2) RNALater RNA stabilization reagent (Qiagen, Venlo, The Netherlands) 3) inflation and 24-hour storage in 10% buffered formalin, change to 70% ethanol and paraffin embedding, and 4) wet/dry lung weight ratio.

1.5 MSC administration during EVLP

Experiments were randomized to control, and $20x10^6$ and $40x10^6$ MSC^{IL-10} groups by TRT, and the team performing EVLP, cell administration, transplantation, and recipient pig care, was blinded to the treatment groups. At the time of EVLP initiation, cryopreserved MSC^{IL-10} were thawed, reconstituted in 20 ml EVLP perfusate solution, and the cell viability was confirmed. Control (20 ml of perfusate solution without $MSCs^{IL-10}$), or $MSCs^{IL-10}$ were administered with an opaque syringe over 20 seconds through an extension tubing attached to the pulmonary artery cannula, followed by a 20 ml perfusate flush. Cell administration was performed after full perfusion flow, normothermia and lung

ventilation had been achieved, and the 1-hour lung assessment and a baseline BAL sample followed by lung recruitment with up to peak airway pressure 25 mmHg for three breaths, had been performed.

1.6 Single-lung transplant survival model

After EVLP, the left lung was transplanted to a recipient pig as described in detail previously.[2] Briefly, tunneled central venous and arterial lines were inserted to the left internal jugular vein and left carotid artery, respectively, left thoracotomy and pneumonectomy were performed, and the left lung was transplanted with a standardized 1-hour warm ischemia time. One hour after reperfusion, blood samples taken selectively from the upper and lower pulmonary veins and the P/F ratios were determined, a lung tissue sample was obtained from the lingula, and the thoracotomy was closed. Arterial blood samples were collected during the recipient recovery phase up to 4 hours, and then taken every 12 hours until the end of the experiment. The recipient pig received medication for ulcer prevention, thromboembolic prophylaxis, antimicrobial prophylaxis and pain management, and intravenous methylprednisolone 1 mg/kg twice daily and peroral cyclosporine A 10 mg/kg twice daily were given as background immunosuppression.[2] All animals received humane care in compliance with the "Principles of Laboratory Animal Care," formulated by the National Society for Medical Research, and The Guide for the Care of Laboratory Animals, published by the National Institutes of Health. The experimental protocol was approved by the Animal Care Committee of the Toronto General Hospital Research Institute (AUP 2999). Details of the

baseline characteristics of donor and recipient pigs, and n-values for each experimental stage, are given in Table S1.

1.7 Lung function evaluation 3 day after transplantation

The recipient pig was anesthetized and tracheostomized 3 days after transplantation, bronchoscopic airway cleaning and 2 three-second lung recruitments with peak airway pressure at 25 mmHg were performed, and systemic and transplant upper and lower vein blood samples for P/F ratio evaluation were obtained using ventilatory settings of pressure control 15 mmHg, PEEP 5 mmHg and FiO_2 1.0. Subsequently, the right pulmonary artery and airways were clamped, and tidal volume, compliance, pulmonary artery pressures, and P/F ratio were determined selectively from the transplant. Finally, BAL was taken from the transplant lower lobe, the recipient was exsanguinated, and tissue samples were taken from the transplant, contralateral native lung, spleen, and various other recipient tissues, and processed as in the EVLP section above.

1.8 Histological evaluation

Lung transplant tissue sections taken 3 days after transplantation were stained with hematoxylin and eosin by the STARR Pathology Core (University Health Network, Toronto, Canada). The histological sections were evaluated for acute lung injury by a pulmonary and transplant pathologist (D.M.H). Each section was scored to assess air space haemorrhage (presence of red blood cells in alveoli), vascular congestion (>75% of alveolar septum occupied with red blood cells), edema/fibrin in the alveoli and presence

of infiltrating white blood cells. These criteria were graded on a scale ranging from normal appearance (0%), mild (<10%), moderate (10-50%) and severe (>50%) abnormalities and scored from 0 to 3, respectively.

1.9 Multiplex fluorescence *in situ* hybridization and immunofluorescence staining

Formalin-fixed paraffin-embedded tissue sections were deparaffinized and antigen retrieval was performed using boiling 0.01M citrate buffer (pH 6) for 20 min. Sections were then washed with water and hybridized with biotinylated ALU probe (Invitrogen, Waltham, MA; Cat. q151P.0100) for 3 hours. After washing with TBS buffer and blocking with DAKO serum-free blocking medium (Agilent DAKO, Santa Clara, CA; Cat. X0909) for 30 min and Avidin/Biotin Blocking Kit (Vector Laboratories, Burlingame, CA; Cat. SP-2001) for 15 min, mouse HLA Class I antibody (Abcam plc, Cambridge, UK; Cat. ab70328) at 1:100 dilution and rabbit Anti-DDDDK tag (binds to FLAG sequence) antibody (Abcam, Cat. ab205606) at 1:50 dilution was incubated overnight for the ALU/HLA Class I/FLAG triple staining. For ALU/CD31/Pan cytokeratin triple staining, rabbit CD31 antibody (Abcam, Cat. ab28364) at 1:300 dilution and mouse pan Cytokeratin (Abcam, Cat. ab7753) at 1:100 dilution was incubated overnight. Subsequently, the sections were washed with PBS-Tween and incubated with Alexa Fluor® 488 Streptavidin (Invitrogen, Cat. S32354), goat anti-rabbit IgG Alexa Fluor® 555 (Invitrogen, Cat. A32732) and goat anti-mouse IgG Alexa Fluor® 647 (Invitrogen, Cat. A21235) for 1 hour at room temperature, washed and counterstained with DAPI.

1.10 Quantitative immunoassays

Perfusate, BAL and lung human IL-10 levels were determined with Human IL-10 Quantikine ELISA Kit (R&D Systems Inc.). Perfusate pig cytokines were determined with Quantikine Elisa Kits for (Porcine IL-1 beta/IL-1F2, IL-6, CXCL8/IL-8 and IL-10 Quantikine ELISA Kits, R&D Systems Inc).

1.11 RNA extraction and quantitative RT-PCR

Lung tissue samples collected in RNAlater were stored at -80℃. RNA was extracted using RNA mini kit (Qiagen) with extra on column DNase treatment. 1000 ng RNA were converted into cDNA using iScript™ Advanced cDNA Synthesis Kit (Bio-Rad Laboratories, Hercules, CA). cDNA was diluted 10 times with water. qPCR was performed on CFX384 Real-Time system (Bio-Rad) using SsoAdvanced™ Universal SYBR® Green Supermix (Bio-Rad) and primers. The relative quantification in gene expression were normalized to internal control and the fold changes were analyzed using delta-delta Ct method. Most of the primers designed were across the intron of the target gene:

Target Gene	Forward Primer (5'-3')	Reverse Primer (5'-3')
IFN-α	TCAGCTGCAATGCCATCTG	AGGGAGAGATTCTCCTCATTTGTG
IFNγ	CCTAAAGGACTATTTTAATGC	TTTTGTCACTCTCCTCTTTCC
Granzyme A	TGCCCCATTCTGCTCTTTGT	AGACTCTTTGCTCTGCCACC
Granzyme B	AAGAGAGCAAGTGGGGAGCG	CTCATGGCCCCCGATGATCT

Pig IL10	TCATCAATTTCTGCCCTGTG	TGTAGACACCCCTCTCTTGGA
IL8	GCTCTCTGTGAGGCTGCAGTT	TTTATGCACTGGCATCGAAGTT
IL1α	AGTTTCCACGCGGAGAGTGA	AAGTGGCTCATAGCTGGCATC
IL1β	GTGATGGCTAACTACGGTGACAA	CTCCCATTTCTCAGAGAACCAAG
IL6	GTCGAGGCTGTGCAGATTAGT	TTCTGTGACTGCAGCTTATCC
TNFα	CCCAAGGACTCAGATCATCG	ATACCCACTCTGCCATTGGA
IL2	GCTCTGGAGGGAGTGCTAAAT	AACAGCAGTTACTGTCTCATCAT
IL17	ACTCCAAACGCTTCACCTCA	TCAGCATTGATACAGCCCGA
Perforin	AATGTGCAGGTGACCGTGGCG	TGAACTCAGGGTGGAGCGGG
IL4	CCCAACCCTGGTCTGCTTAC	TTCTCCGTCGTGTTCTCTGG
Ppia	CCCACCGTCTTCTTCGACAT	CCTTTCTCCCCAGTGCTCAG
GAPDH	TCGGAGTGAACGGATTTGGC	TGACAAGCTTCCCGTTCTCC

1.12 Quantitative ALU PCR analysis

MSCs were assessed using quantitative PCR for noncoding human ALU repeats, as described previously.[5] Briefly, genomic DNA was extracted from pig lung, liver, kidney, heart, spleen and lymphoid tissues using DNeasy Blood and Tissue kit (Qiagen). The sequence of the PCR primers and the probe used for detection of human ALU repetitive sequences were as follows: Alu forward, 5'-CAT GGT GAA ACC CCG TCT CTA-3'; Alu reverse, 5'-GCC TCA GCC TCC CGA GTA G-3'; TaqMan probe, 5'-FAM-ATT AGC CGG GCG TGG TGG CG-TAMRA-3' (Integrate DNA Technologies, Coralville, Iowa). PCR assays for ALU sequences were performed in a volume of 10 µl that contained 5 µl of

PrimeTime Gene Expressio Master Mix, 900 nM each of the forward and reverse primers, 250 nM TaqMan probe, and 500 ng of target template. Reactions were incubated at 95°C for 3 min to activate the polymerase followed by 40 cycles at 95°C for 15 s and 60°C for 1 min. Standard curves were generated by serial dilution of human genomic DNA prepared from $1x10^6$ MSCs into samples spiked with 500 ng pig genomic DNA. Results were expressed as ng human DNA per 500 ng of pig DNA.

1.3 TUNEL staining and automatic image quantification

Paraffin-embedded lung tissue sections were subjected to TUNEL apoptosis assay (STARR Pathology Core) by with chromogenic detection with diaminobenzidine (DAP). The tissue sections were scanned on Aperio scanners (Leica Biosystems, Wetzlar, Germany) and imported into the HALO software database (Indica Labs, Albuquerque, NM). A Random Forest classifier was trained to detect large airway epithelium within the tissue section by selecting several example regions of interest for the following classes: large airway epithelium, alveolar compartment, and whitespace (regions not containing tissue). Through the classifier, the large airway epithelium was separated from the alveolar compartment such that the two can be analyzed independently of one another. Manual corrections were done to ensure accurate large airway epithelium detection. The classifier was adjusted for each condition to ensure its ability to discriminate between epithelium and stroma accurately and applied to all images within each dataset. Manual QC inspection and was performed following application of the classifier to ensure similar regions were being identified between the various conditions.

Quantitative analysis of the TUNEL biomarker was performed using the Indica Labs - Multiplex IHC v 2.0.3 algorithm. The analysis was done by selecting a brown cell as an example for the DAB stain and a blue cell as an example of the Hematoxylin counterstain. Thresholds were set to identify DAB positive nuclei by the presence of counterstain, separate closely packed cells, and identify thresholds for positivity of TUNEL. The nuclear size was set to detect nuclei between 8.8496 and 146.0177 μm^2. The minimum intensity for nuclei to be considered positive for TUNEL was set to 0.079. The percentage of positive cells in each marker was reported independently for the regions of interest (large airway epithelium and alveolar compartment) for each image analyzed. Data was exported for transfer.

1.14 Flow cytometry

Cryopreserved lung single cell suspension samples were thawed and counted with hemocytometer with trypan blue for live-dead discrimination, and a maximum of $1x10^6$ live cells were obtained from each sample for antibody staining. Samples designated for the T cell panel were incubated with eBioscience™ cell stimulation cocktail (Invitrogen) and GolgiStop™ (BD Biosciences) at 37°C, 5% CO_2 for 6 hours according to manufacturer instructions. Samples were stained with fixed viability stain 620 (BD Biosciences) according to manufacturer's instructions. Cells were then stained with the following antibodies in this order: unconjugated primary antibody, a fluorochrome-conjugated secondary antibody, a standard panel of fluorochrome-conjugated monoclonal antibodies (Table S2), and a streptavidin stain for 15 minutes at room temperature. Each stain was followed by a wash step with flow cytometry buffer (1x PBS

with 2% v/v heat-inactivated fetal bovine serum and 2mM EDTA). Cells were fixed for intracellular staining, using either the BD Cytofix/Cytoperm kit (BD Biosciences) or eBioscience™ FoxP3/transcription factor staining buffer set (Invitrogen) and stained with fluorochrome-conjugated intracellular antibodies according to kit instructions. Flow cytometric data was acquired on LSR II (BD Biosciences) and compensation performed at the beginning of each experiment. Data were analysed using FlowJo (v10, FlowJo LLC).

1.15 Mixed lymphocyte reaction

Donor and recipient spleen cells were obtained at the time of euthanasia and cryopreserved. Thawed donor and recipient cells were counted, labeled with carboxyfluorescein succinimidyl ester (CFSE, Thermo Fisher Scientific) and eFluor 450 cell proliferation dye (Thermo Fisher Scientific), respectively. Donor cells were irradiated (20 Gy) in a 137Cs irradiator (Best Theratronics). Cells were co-cultured for 5 days in 96-well round bottom tissue culture plates in AIM V media containing 50 units/mL recombinant pig interleukin 2. Cells were then stained with fixable viability stain 700 (BD Biosciences), anti-pig CD4 PE-Cy7 and antipig CD8 PE antibodies prior to acquisition on a Fortessa flow cytometer (BD Biosciences). Data were analyzed with FlowJo (BD Biosciences).

1.16 Statistics

Data are expressed as mean ± standard deviation and analyzed with GraphPad Prism 10.2.1 (GraphPad Software, San Diego, CA). Continuous variables were compared with 2-tailed Student's t-test, or one-way or two-way ANOVA with Dunnett correction, or in case of missing values with mixed-effect analysis with Šidák correction, comparing treatment groups to the control group. Non-continuous parameters were compared with Kruskal-Wallis test with Dunn correction, comparing treatment groups to the control group. $p<0.05$ was considered statistically significant.

References

1. Nykänen AI, Mariscal A, Duong A, et al. Engineered Mesenchymal Stromal Cell Therapy During Human Lung Ex Vivo Lung Perfusion is Compromised by Acidic Lung Microenvironment. *Mol Ther Methods Clin Dev*. 2021:In press.
2. Mariscal A, Caldarone L, Tikkanen J, et al. Pig lung transplant survival model. *Nat Protoc*. Aug 2018;13(8):1814-1828. doi:10.1038/s41596-018-0019-4
3. Cypel M, Yeung JC, Hirayama S, et al. Technique for prolonged normothermic ex vivo lung perfusion. *J Heart Lung Transplant*. Dec 2008;27(12):1319-25. doi:10.1016/j.healun.2008.09.003
4. Yeung JC, Wagnetz D, Cypel M, et al. Ex Vivo Adenoviral Vector Gene Delivery Results in Decreased Vector-associated Inflammation Pre- and Post-lung Transplantation in the Pig. *Molecular Therapy*. 2012;20(6):1204-1211. doi:10.1038/mt.2012.57
5. Munoz JR, Stoutenger BR, Robinson AP, Spees JL, Prockop DJ. Human stem/progenitor cells from bone marrow promote neurogenesis of endogenous neural stem cells in the hippocampus of mice. *Proc Natl Acad Sci U S A*. Dec 13 2005;102(50):18171-6. doi:10.1073/pnas.0508945102

SUPPLEMENTARY FIGURES

Figure S1

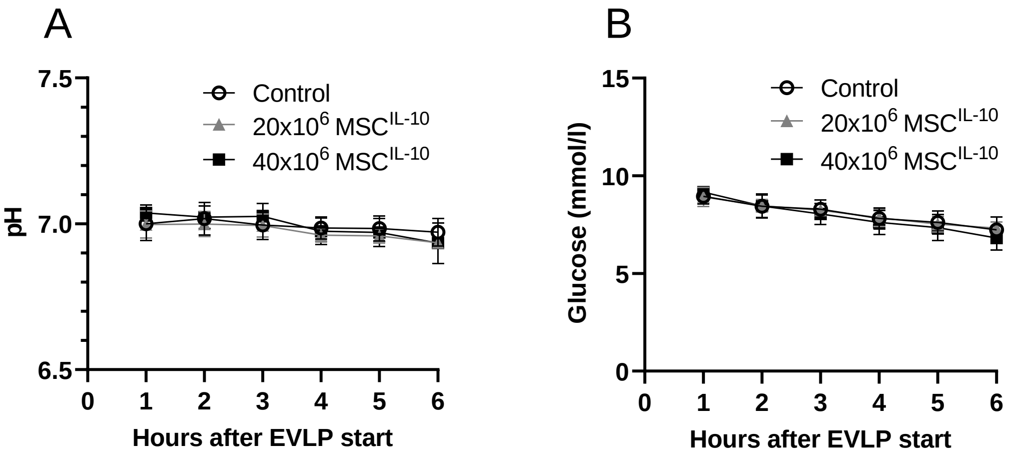

Supplementary Figure 1S. Metabolic parameters during EVLP. Perfusate (A) pH and (B) glucose remained stable during the EVLP in all groups. Data mean±SD, analyzed by 2-way-ANOVA with Dunnett correction comparing treatment groups to the control group. EVLP, ex vivo lung perfusion; IL-10, interleukin-10; MSC, mesenchymal stromal cell.

Figure S2

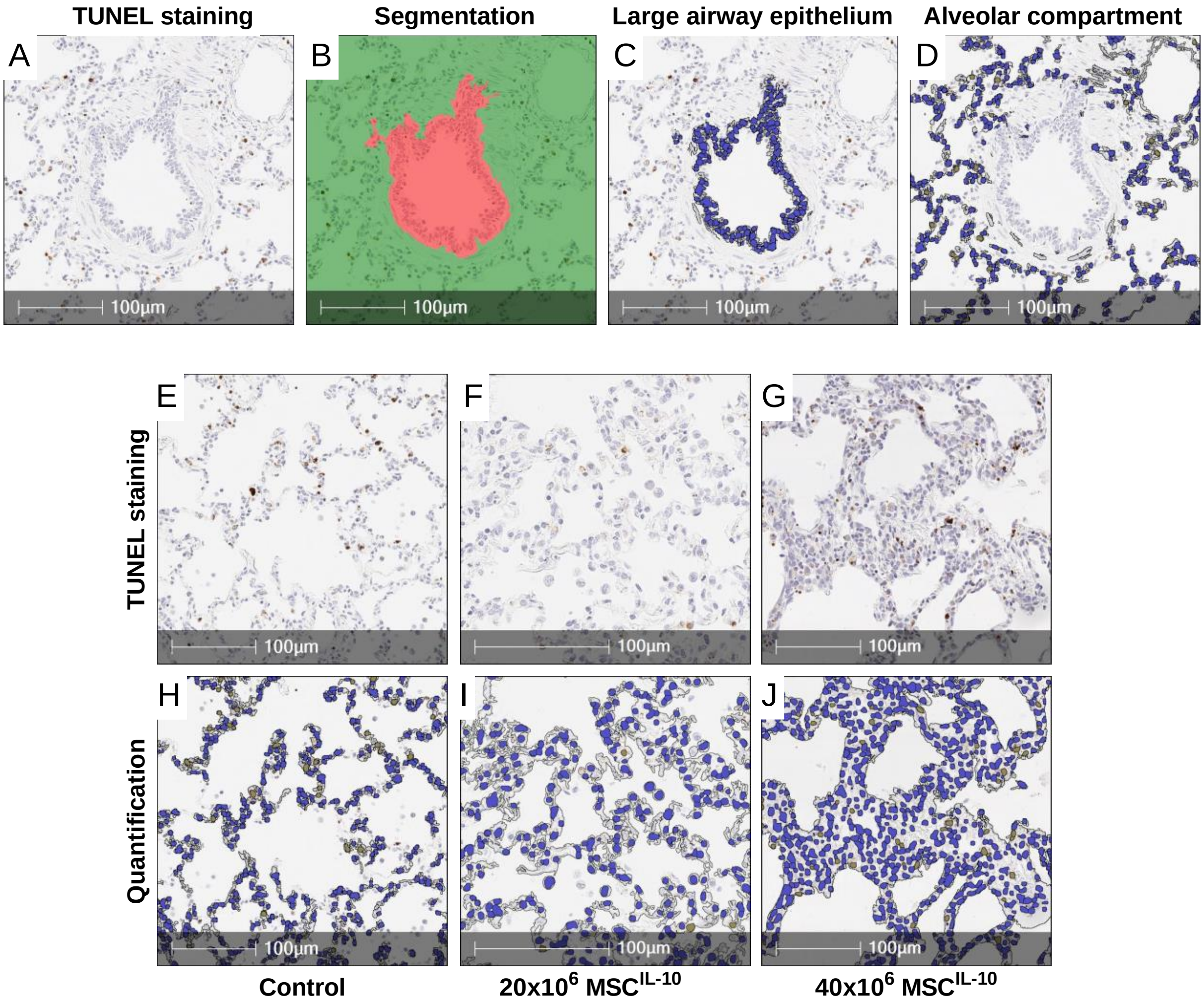

Supplementary Figure S2. TUNEL staining and analysis. Lung paraffin-embedded tissue sections were subjected to TUNEL apoptosis assay with chromogenic detection with DAP (brown signal). The stained sections were scanned and analyzed by image analysis platform using algorithms for automated stromal and epithelial segmentation. Example of tissue segmentation and quantification showing (A) light microscopy image of a TUNEL staining, (B) respective segmentation of large airway epithelium area (red)

and alveolar compartment (green), and (C and D) automated analysis of TUNEL positive (blue) and negative (green) nuclei. Quantification and representative sections of $TUNEL^+$ apoptotic cells in the alveolar compartment of (E and H) control, (F and I) $20x10^6$ and (G and J) $40x10^6$ MSC^{IL-10} lungs 6 hours after EVLP start. DAP, diaminobenzidine; EVLP, ex vivo lung perfusion; IL-10, interleukin-10; MSC, mesenchymal stromal cell, TUNEL, deoxynucleotide transferase-mediated deoxy uridine triphosphate nick-end labeling.

Figure S3

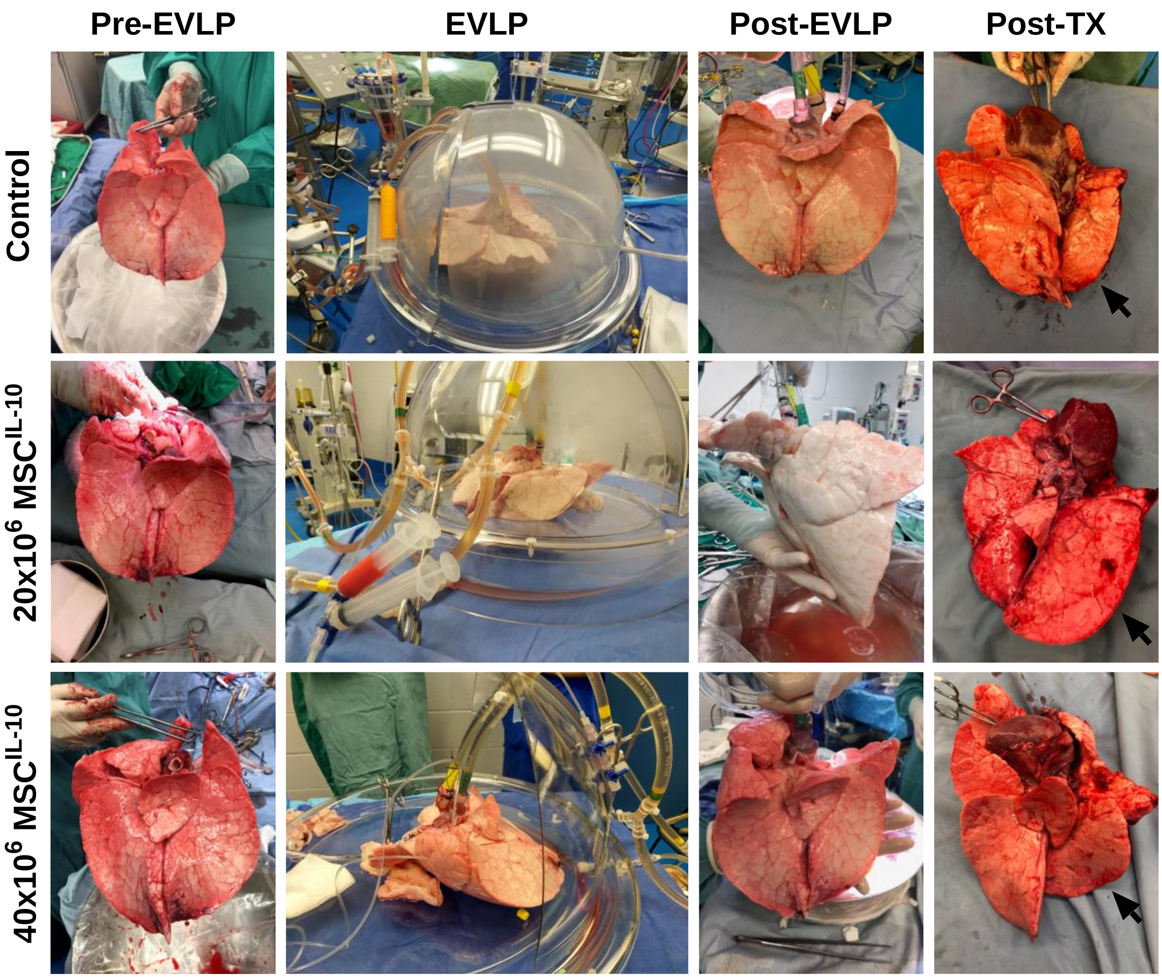

Supplementary Figure S3. Macroscopic lung appearance during the experiment. Macroscopic appearance of double lungs before, during and after EVLP, and 3 days after left single lung transplantation in control, $20x10^6$ and $40x10^6$ MSC^{IL-10} groups. Black arrows point to the transplanted left lung.

TUNEL staining and analysis. Lung paraffin-embedded tissue sections were subjected to TUNEL apoptosis assay with chromogenic detection

Figure S4

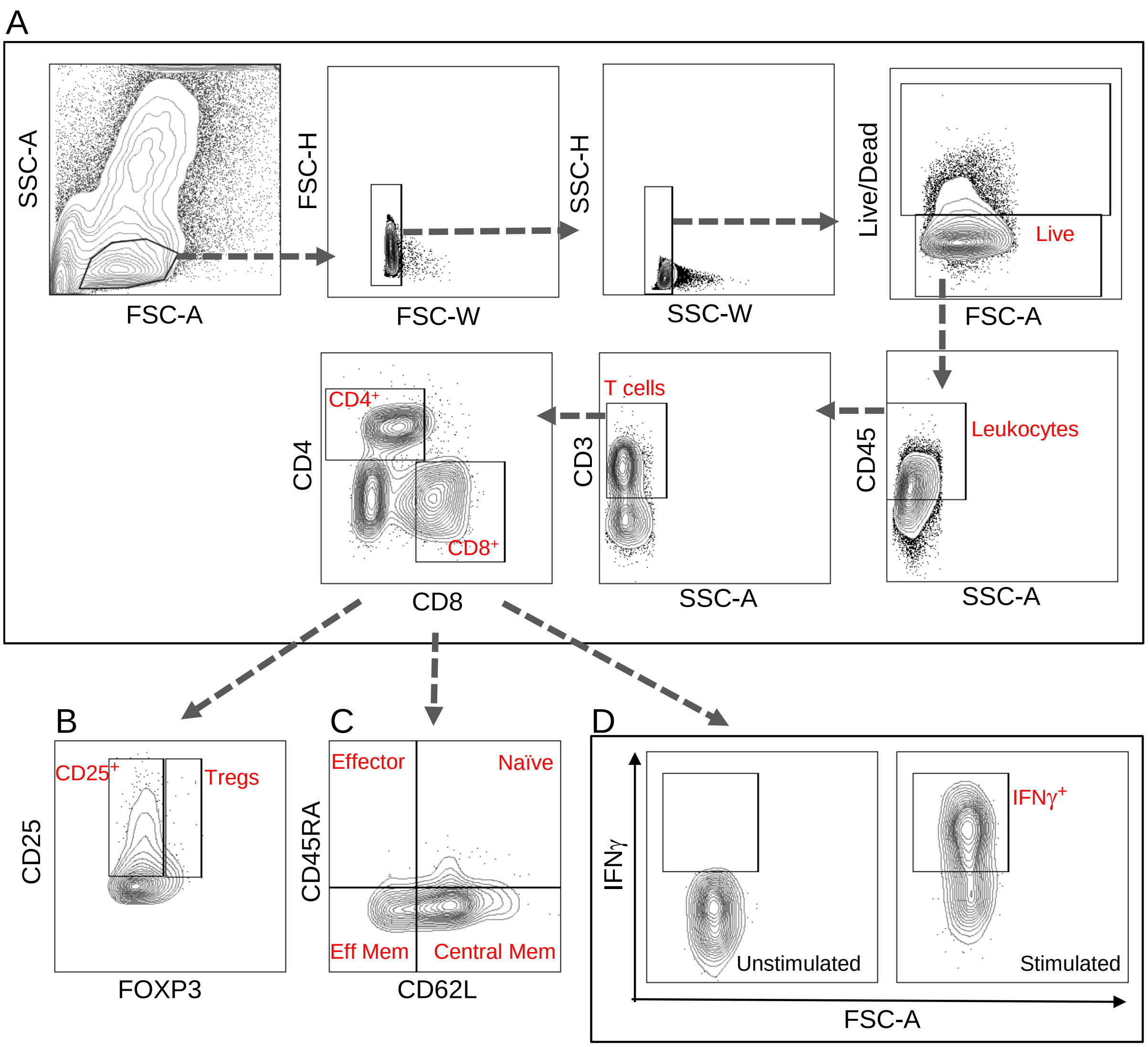

Supplementary Figure S4. T cell flow cytometry gating strategy and analysis. (A) Debris, large cells and doublets were gated out and lung T cells identified by pan-leukocyte marker CD45 and T cell markers CD3, CD4, and CD8. T cells were further subclassified according to their (B) CD25 and FOXP3, and (C) CD45RA and CD62L expression, and (D) IFNγ expression was determined in unstimulated and stimulated cells. IFN, interferon.

Figure S5

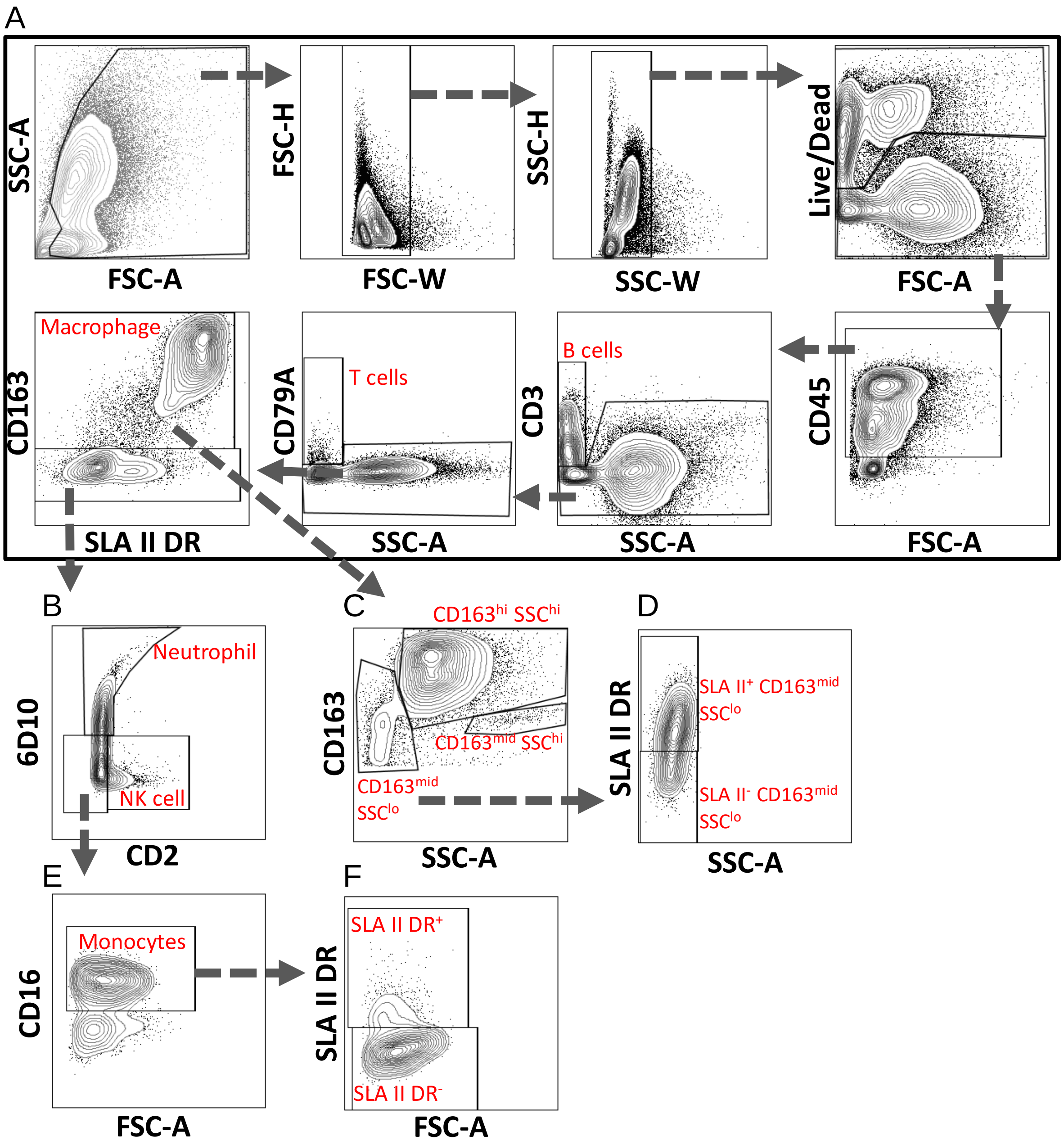

Supplementary Figure S5. Myeloid cell flow cytometry gating strategy and analysis. (A) Debris and doublets were gated out and lung myeloid cells were identified by pan-

leukocyte marker CD45, and exclusion of $CD3^+$ T cells and $CD79a^+$ B cells. (B) Neutrophils were identified by marker 6D10 and NK cells by CD2 expression, (C) macrophage subtypes by CD163 expression level and granularity, (E) and monocytes by CD16 expression. (D and F) $CD163^{mid}SSC^{low}$ macrophages and $CD16^+$ monocytes were further classified with pig MHC (SLA) class II DR expression. MHC, major histocompatibility complex; NK, natural killer.

Figure S6

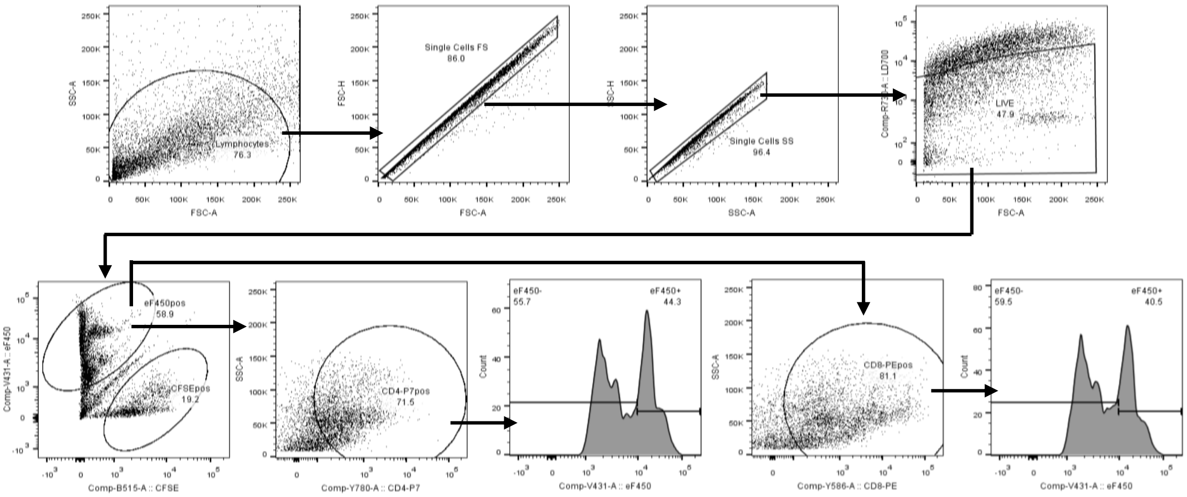

Supplementary Figure S6. Mixed lymphocyte reaction cell proliferation flow cytometry gating strategy and analysis. Shown is the gating strategy to include viable $CD4^+$ and $CD8^+$ cells labelled with the eFluor cell proliferation dye.

SUPPLEMENTARY TABLES

Table S1. Baseline parameters

	Control	20x10^6 MSC^{IL-10}	40x10^6 MSC^{IL-10}	p-value 20x10^6 MSC^{IL-10}	p-value 40x10^6 MSC^{IL-10}
Donor					
n=	7	5	6		
Weight, kg	34.3±1.6	32.7±1.4	33.9±2.1	0.24	0.87
P/F ratio, mmHg	530±42	484±45	496±116	0.48	0.64
Cold ischemia time, h	24.3±0.2	24.5±0.2	24.2±0.3	0.52	0.42
EVLP 1-hour (pre-MSC) parameters					
n=	7	5	6		
PAP, mmHg	8.6±1.9	10.4±1.9	8.8±1.5	0.18	0.95
PVR, dynes/sec/cm−5	244±103	340±110	272±74	0.19	0.83
Peak airway pressure, cmH2O	18.9±4.3	17.4±2.2	18.0±3.3	0.72	0.88
Mean airway pressure, cmH2O	6.9±0.7	6.8±0.4	7.2±0.4	0.98	0.51
Dynamic compliance, ml/cmH2O	19.7±6.2	20.4±3.2	21.5±5.6	0.96	0.78
Static compliance, ml/cmH2O	23.6±6.6	24.7±5.2	24.7±5.2	0.92	0.92
Delta PaO2, mmHg	276±79	299±63	255±70	0.82	0.82
MSC treatment					
n=	0	5	6		
MSC administration after thawing, min		67±4	73±3	NA	NA
MSC administration after EVLP start, min		78±1	81±4	NA	NA
MSC viability, %		92.2±1.3	90.5±3.1	NA	NA
MSC hIL-10 production in vitro, pg/cell/h		0.23±0.03	0.23±0.03	NA	NA
Lung transplant					
n=	6	5	4		
Reason for no transplantation	No recipient available (n=1)		Recipient died during anesthesia induction (n=1)		
Recipient weight, kg	34.1±1.5	32.5±2.7	34.0±1.3	0.3	1
Recipient pre-transplant P/F ratio, mmHg	501±56	507±33	442±42	0.98	0.23
Warm ischemia time, min	57±6	56±8	52±6	0.93	0.49
3-day survival, n	5	5	3		
Reasons for recipient loss before POD 3	Arterial-line related stroke POD 1 unrelated to lung function		Arterial-line related bleeding POD 0 (5h) unrelated to lung function		

EVLP, Ex Vivo Lung Perfusion; IL-10, interleukin-10; P/F ratio, ratio of arterial oxygen partial pressure to fractional inspired MSC, mesenchymal stromal cell; PAP, pulmonary artery pressure; POD, postoperative day; PVR, pulmonary vascular resistance. Data mean±standard deviation, analyzed by 1-way ANOVA with Dunnett's correction comparing treatment groups to the control group

Table S2. Flow cytometry antibodies.

Antibody	Clone	Isotype	Conjugate	Company	Catalog #
**T cell Panel**					
CD3	BB23-8E6-8C8	Mouse IgG2a	PerCP-Cy5.5	BD Biosciences	561478
CD4	74-12-4	Mouse IgG2b	PE-Cy7	BD Biosciences	561473
CD8	74-2-11	Mouse IgG2a	PE-Cy5	abcam	ab25536
CD25	K231.3B2	Mouse IgG1	Pure	BioRad	MCA1736GA
CD44	IM7	Rat IgG2b	BV605	BioLegend	103047
CD45	K252.1E4	Mouse IgG1	AF647	BioRad	MCA1222A647
CD45RA	MIL13	Mouse IgG1	FITC	BioRad	MCA1751F
CD62L	SK11	Mouse IgG2a	BV711	BD Biosciences	565040
FoxP3	FJK-16s	Rat IgG2a	AF700	eBioscience	56-5773-82
IFNγ	CC302	Mouse IgG1	PE	BioRad	MCA1783PE
Secondary Antibody	Polyclonal	Goat IgG (H+L)	BV421	Jackson ImmunoResearch Laboratories	115-675-146
**Myeloid panel**					
6D10	6D10	Mouse IgG2a	Pure	BioRad	MCA2599GA
CD2	RPA-2.10	Mouse IgG1	PE-Cy5	BioRad	MCA2814C
CD3	BB23-8E6-8C8	Mouse IgG2a	PerCP-Cy5.5	BD Biosciences	561478
CD16	FcG7	Mouse IgG1	Biotin	BD Biosciences	551395
CD45	K252.1E4	Mouse IgG1	AF647	BioRad	MCA1222A647
CD79a	HM47	Mouse IgG1	APC-eFluor 780	Invitrogen	47-0792-42
CD163	2A10/11	Mouse IgG1	PE	BioRad	MCA2311PE
SLA II DR	2E9/13	Mouse IgG2b	FITC	BioRad	MCA2314F
Secondary Antibody	Polyclonal	Goat IgG (H+L)	BV421	Jackson ImmunoResearch Laboratories	115-675-146

Streptavidin	BV605	BD Biosciences	563260
